# Early and Ultraearly Administration of Tranexamic Acid in Traumatic Brain Injury: Our 8-Year-Long Clinical Experience

**DOI:** 10.1155/2020/6593172

**Published:** 2020-09-18

**Authors:** Nurdan Acar, Mustafa Emin Canakci, Ugur Bilge

**Affiliations:** ^1^Emergency Department, School of Medicine, Eskisehir Osmangazi University, Eskisehir, Turkey; ^2^Family Medicine Department, School of Medicine, Eskisehir Osmangazi University, Eskisehir, Turkey

## Abstract

**Introduction:**

The most important result of head trauma, which can develop with a blunt or penetrating mechanism, is traumatic brain injury. Tranexamic acid (TXA) can be used safely in multiple trauma. Recent studies showed that TXA can be useful in management of intracerebral hemorrhage, especially in reducing the amount of bleeding. The TXA given in the first 3 hours has been shown to reduce mortality. The aim of our study was to evaluate the effectiveness of tranexamic acid used in patients with traumatic brain injury.

**Method:**

Patients with trauma in the emergency room between January 2012 and January 2020 were screened in this retrospective study. The inclusion criteria were being over the age of 18 years, tranexamic acid administration in the emergency department, and traumatic brain injury on brain computerized tomography (CT) and control CT imaging after 6 hours.

**Results:**

The number of study patients was 51. The median score of GCS was 12.00 (8.00–15.00). Subdural hemorrhage and subarachnoid hemorrhage were the most common findings on brain CT scans. In the group TXA treatment for less than 1 hour, the arrival MAP was low and the pulse was high (*p*=0.022 and *p*=0.030, respectively). All the patients were admitted with multiple trauma. None of the 51 patients had thrombotic complications and died due to head injury.

**Conclusion:**

TXA appears to be a safe drug with few side effects in the short term in head injuries. According to our experience, it comes to mind earlier in multiple trauma, especially in head trauma with pelvic trauma.

## 1. Introduction

The most important result of head trauma that can occur with blunt or penetrating mechanism is traumatic brain injury (TBI). It has been reported in the literature that there are an average of 1.1 million head trauma applications to emergency departments (ED) each year. It was stated that 21% of these cases required hospitalization and 4% were exitus [1].

In studies conducted, the risk of bleeding due to head trauma was found to be approximately 3-4 times higher in patients over 50 years of age than in those under 30 years old. It has been reported that 15.7% of mild head injuries require hospitalization and 35% of these hospitalizations included patients of65 years and older [2]. Even mild head traumas have been reported to cause mortality and serious morbidity in patients using antiaggregants or anticoagulants [3, 4]. Secondary brain injury from intracranial bleeding, cerebral edema, and increased intracranial pressure is the cause of morbidity and mortality after TBI [4, 5].

The antifibrinolytic agent tranexamic acid (TXA) is commonly given to patients to reduce the need for blood transfusion. TXA reduces the number of patients receiving a blood transfusion by about one-third, reduces the volume of blood transfused by about one unit, and halves the need for further surgery to control bleeding in elective surgical patients [6]. TXA has been shown to reduce mortality in trauma patients with extracranial bleeding. The CRASH-2 trial showed that the administration of TXA within 8 h of injury significantly reduces deaths due to bleeding compared to placebo, with no apparent increase in vascular occlusive events [7]. Studies have shown that tranexamic acid can be used safely in head trauma and in the management of intracerebral hemorrhages, especially in reducing the amount of bleeding [8, 9]. The TXA given in the first 3 hours has been shown to reduce mortality, and the regimen of starting treatment and maintaining it in 8 hours is used in the early period [7].

The aim of our study was to evaluate the effectiveness of tranexamic acid used in patients with traumatic brain injury.

## 2. Materials and Methods

This study was conducted retrospectively in a tertiary university hospital with an average of 100,000 emergency applications per year. Patients who came to the emergency room with trauma between January 2012 and January 2020 were screened. The inclusion criteria of being over the age of 18 years, TXA administration in the emergency department, and traumatic brain injury on computed brain tomography were employed. Patients who underwent computerized tomography 6 hours after formed the study group. Patients who are given TXA in the emergency department are started with 1 gr IV in 10 minutes, and then, a 1 gr maintenance regimen is applied in 8 hours. As it is an observational study, it is arranged according to the STROBE statement.

By evaluating the patient's file information, age, gender, trauma mechanism, mean arterial pressure, pulse, oxygen saturation, drugs used, Glasgow Coma Scale (GCS), pupil reflex, pH, lactate, base deficit, brain computerized tomography (CT) findings, bleeding in other regions tomography, TXA administration time, control brain CT time, and increased bleeding in control brain CT were evaluated. In the files, the increase in bleeding in the radiology reports and related clinical notes is noted. The increase in bleeding was evaluated as an enlargement over 1 mm in BT, increase in shift, and any increase in bleeding volume.

### 2.1. Statistical Analysis

Continuous data are given as mean ± standard deviation. Categorical data are given as percentage (%). Shapiro–Wilk's test was used to investigate the suitability of the data for normal distribution. In comparison to groups that do not conform to normal distribution, the Mann–Whitney *U* test was used for cases with two groups. Pearson chi-square and Pearson exact chi-square analyzes were used in the analysis of the created cross tables. Logistic regression analysis was used to determine risk factors. IBM SPSS Statistics 21.0 (IBM Corp. Released 2012. IBM SPSS Statistics for Windows, Version 21.0, Armonk, NY: IBM Corp.) program was used in the implementation of the analyzes. For statistical significance, *p* < 0.05 value criterion was accepted.

## 3. Results

**Figure 1:**
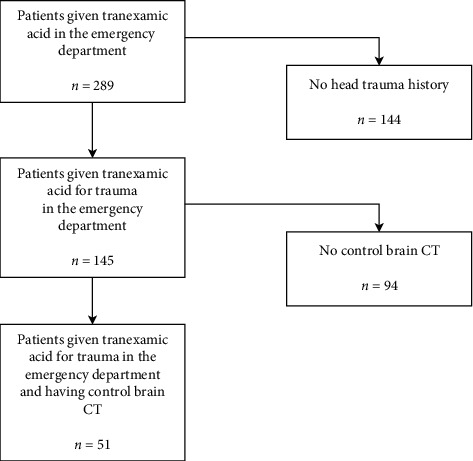
Flow diagram of the study population.

The number of patients receiving TXA in the emergency room was determined to be 289. 145 of them were patients with trauma. The number of patients with TBI in the emergency department, TXA given for any reason and control brain CT after hospitalization, was determined as 51 (Figure 1).

The median age of the patients was 44.00 (32.00–66.00). The number of female patients was determined as 13 (25.5%). Trauma mechanisms, vital parameters, and demographic features are given in Table 1. The most common injury was *In-Vehicle Traffic Accident*. When the patients who were at risk of bleeding from the drugs used by the patients were evaluated, the most used drug was found to be acetylsalicylic acid (ASA). Three patients were using clopidogrel in the study group. Other antiaggregant and anticoagulant medication uses were not detected.

Neurological examination evaluations and laboratory findings of the patients are given in Table 2. The median value of GCS was 12.00 (8.00–15.00) in the study group. 41 (80.4%) of the patients had pupil reaction.

The tomography findings of the study group are given in Table 3. In the study group CT, subdural hemorrhage and subrachnoid hemorrhage were the most common on brain CT. It was found that hemothorax and pelvic bleeding were higher in thorax, abdominal, and pelvic CT, where other injuries were evaluated. An increase in the amount of bleeding in control tomography was observed in 8 patients in the study group. The increase in control tomography was evaluated as an enlargement of more than 1 mm and any volume increase.

Since TXA was given in our center within 3 hours, no patient received TXA after 3 hours. 26 (51.0%) of the patients received TXA in the first hour. The characteristics of the patients whose TXA-taking time periods are evaluated are given in Table 4. In patients with low MAP and high pulse, TXA was thought to be preferred earlier (*p* = 0.022 and *p* = 0.030, respectively). When the duration of TXA administration was evaluated with CTs taken, it was observed that TXA treatment was given earlier in patients with pelvic injury (*p* = 0.022). It is thought that physicians act earlier, since bleeding can be detected late in the case of pelvic injury, and there is no mechanism to stop bleeding in this area.

All of the patients were admitted with multiple trauma. None of the 51 patients had thrombotic complications and died due to head injury. In the case of isolated head injury, it should be considered that it can be used safely as in multiple trauma.

## 4. Discussion

TXA is a safely used agent especially in the management of multitrauma patients. Due to decrease in the amount of bleeding, morbidity, and mortality, as a result of its increasing use in the last decade, bleeding-related deaths (exsanguination) decrease [8, 10]. The use of TXA in combination with performing damage control surgery, proper use of blood products, and thromboelastography has an important role in this reduction. The advantages of TXA are that it has no obvious contraindications, it is inexpensive, it is given only by iv infusion, and its complications are rare [11–13]. In our study, we wanted to evaluate the benefit of TXA for intracranial hemorrhage in patients with multiple trauma, however, with TBI. In the CRASH-3 study published in 2019, the benefits of using TXA in mild head injuries were mentioned [8]. In our study, we found that there were no patients who died due to intracranial hemorrhage in the early period, especially after TXA intake. Since our study is retrospective, it should be noted that TXA was not given to patients with isolated TBI before the CRASH-3 study was published.

In our study, the median age was determined to be 44, and a rate similar to the average of 41.7 in the CRASH-3 study was achieved, in which most patients were studied. The fact that trauma especially affected the young population was the most important factor in this regard. In our study, females were more affected than other studies [8, 10]. The CRASH-3 showed the efficacy of the TXA in reducing the mortality rate only on mild and moderate TBI and not on severe TBI. Our sample size was small, and we could not compare the mild or severe TBI differences about TXA.

In our study, the duration of TXA administration was compared between 1 hour and 1–3 hours. Previous studies have shown that with the onset of TXA at the appropriate time, all-cause deaths are reduced and hospital stay is shorter [10, 14]. In the CRASH-2 study, all-cause mortality rates were 14.5% in the group of patients receiving TXA and 17.4% in the MATTERs study. Although we could not compare in our study that there was no control group, all-cause mortality rates were 18%, similar to other studies [8, 10, 15].

In the CRASH-2 study published in 2010, evaluating the use of TEA in 20.211 trauma patients with hemorrhage or hemorrhagic shock, a significant decrease in mortality due to all causes and a decrease in bleeding-related mortality were also detected. Moreover, TXA exhibited these positive effects without causing any vascular occlusion or thrombosis [7]. The MATTERs study, designed similar to the CRASH-2 study and performed in the military field, showed that mortality was reduced in polytrauma patients treated with TXA; such reduction was more pronounced in those undergoing “massive” transfusions of blood products, including packed red blood cells, fresh frozen plasma, platelets, and cryoprecipitates to restore circulating volume and clotting factors [15].

Our study group is small-sized and gunshot wounds were seen in only two patients. But gunshot wounds are known for their devastating consequences in terms of profuse intracranial bleeding; hence, TXA can be useful [16].

TXA research has been performed in many bleeding forms due to the variety of ways of using the drug and still continues [17]. In observational studies in which IVA was performed by applying IV, mortality rates were found lower than in the current literature, but no comparison was made because there was no control group [18, 19].

Although there are articles about the restriction of the use of TXA maintenance therapy in recent years, we found that maintenance therapy did not cause any complications in our study [13, 20].

TXA may increase the risk of thromboembolism: in the MATTERs study, the rates of deep vein thrombosis and pulmonary embolism were greater in the TXA group than in the no-TXA group [15]. There are several studies about thromboembolic complications in neurosurgery [21, 22], and these indicate that the perioperative period of immobility should be tackled with appropriate thromboprophylaxis protocols. It should also be highlighted that strict monitoring of coagulation cascade (platelet count, INR, PT, and PTT) is helpful in the acute phase of their management [23, 24].

## 5. Conclusion

In light of the above, TXA appears to be a safe drug with few side effects in the early management of TBI. According to our experience, its use is particularly helpful in multiple trauma or whenever TBI is associated to pelvic trauma. Further studies are warranted to establish the role of TXA in the ED management of patients with isolated and nonisolated TBI.

## Figures and Tables

**Table 1:** Demographic data, trauma mechanisms, and medications of the patients.

	
Age (years)	44.00 (32.00–66.00)

*Sex*	
Female (*n* (%))	13 (25.5%)

*Vitals*	
MAP (mmHg)	83.33 (66.67–93.33)
Pulse (bpm)	88.00 (85.00–110.00)
Saturation (%)	95.00 (91.00–97.00)

*Trauma mechanism*	
MVA (*n* (%))	17 (33.3%)
Pedestrian trauma (*n* (%))	9 (17.6%)
Motorcycle bike (*n* (%))	5 (9.8%)
Fall from heights (*n* (%))	16 (31.4%)
Assault (*n* (%))	1 (2.0%)
Gunshot injuries (*n* (%))	2 (3.9%)
Stab wounds (*n* (%))	1 (2.0%)

*Medications*	
ASA (*n* (%))	16 (31.4%)
Clopidogrel (*n* (%))	3 (5.9%)

MAP, mean arterial pressure; MVA, motor vehicle accident; ASA, acetylsalicylic acid.

**Table 2:** Neurological examination and laboratory findings.

*Neurologic exam*	
GCS	12.00 (8.00–15.00)
Pupil reaction	41 (80.4%)

*Laboratory*	
pH	7.364 (7.284–7.398)
Lactate (mmol/L)	3.90 (2.40–4.90)
Base deficit (mmol/L)	−4.20 (−7.10–−2.20)

GCS, Glasgow Coma Scale.

**Table 3:** CT findings of the study population.

	Study
*Brain CT findings*	
Contusion (*n* (%))	23 (45.1%)
Subarachnoid hemorrhage (*n* (%))	21 (41.2%)
Subdural hematoma (*n* (%))	22 (43.1%)
Epidural hematoma (*n* (%))	10 (19.6%)
Intraparenchymal (*n* (%))	4 (7.8%)
Other (pneumocephalus) (*n* (%))	2 (3.9%)
Total (*n* (%))	

*Other injuries*	
Hemothorax (*n* (%))	9 (17.6%)
Liver injury (*n* (%))	5 (9.8%)
Splenic injury (*n* (%))	3 (5.9%)
Kidney injury (*n* (%))	2 (3.9%)
Pelvic bleeding (*n* (%))	11 (21.6%)
External bleeding (*n* (%))	3 (5.9%)
*Control CT time* (Δ hours)	14.00 (7.00–26.50)
*Control CT increased bleeding* (*n* (%))	8 (15.7%)

CT, computerized tomography.

**Table 4:** The factors affecting the time of TXA treatment.

	TXA <1 h	TXA 1–3 h	*p*
Age (years)	45.00 (25.75–66.00)	42.00 (33.00–66.50)	0.970
Female (*n* (%))	6 (23.1%)	7 (28.0%)	0.687^*∗*^
MAP (mmHg)	73.33 (63.33–90.83)	90.00 (75.00–95.00)	**0.022**
Pulse (bpm)	100.00 (90.00–110.00)	90.00 (79.00–102.50)	**0.030**
Saturation (%)	92.50 (90.00–95.25)	95.00 (91.50–97.50)	0.067
GKS	12.00 (7.00–14.25)	12.00 (8.50–15.00)	0.871
pH	7.356 (7.275–7.391)	7.365 (7.318–7.405)	0.361
Lactate (mmol/L)	3.70 (2.40–4.90)	3.90 (2.25–5.10)	0.977
Base deficit (mmol/L)	−5.10 (−8.13–−2.82)	−3.50 (−6.35–−1.75)	0.184

^*∗*^Pearson chi-square. p  <  0.05 is statistically significant.

## Data Availability

The data used to support the findings of this study are available from the corresponding author upon request.
